# Genome-resolved metagenomics provides insights into the ecological roles of the keystone taxa in heavy-metal-contaminated soils

**DOI:** 10.3389/fmicb.2023.1203164

**Published:** 2023-07-21

**Authors:** Liangzhi Li, Delong Meng, Huaqun Yin, Teng Zhang, Yongjun Liu

**Affiliations:** ^1^School of Minerals Processing and Bioengineering, Central South University, Changsha, China; ^2^Key Laboratory of Biometallurgy of Ministry of Education, Central South University, Changsha, China; ^3^Hunan Urban and Rural Environmental Construction Co., Ltd, Changsha, China; ^4^Hunan Tobacco Science Institute, Changsha, China

**Keywords:** heavy metal resistance, metagenome-assembled genomes, contaminated soils, keystone taxa, metagenomics

## Abstract

Microorganisms that exhibit resistance to environmental stressors, particularly heavy metals, have the potential to be used in bioremediation strategies. This study aimed to explore and identify microorganisms that are resistant to heavy metals in soil environments as potential candidates for bioremediation. Metagenomic analysis was conducted using microbiome metagenomes obtained from the rhizosphere of soil contaminated with heavy metals and mineral-affected soil. The analysis resulted in the recovery of a total of 175 metagenome-assembled genomes (MAGs), 73 of which were potentially representing novel taxonomic levels beyond the genus level. The constructed ecological network revealed the presence of keystone taxa, including *Rhizobiaceae, Xanthobacteraceae, Burkholderiaceae*, and *Actinomycetia*. Among the recovered MAGs, 50 were associated with these keystone taxa. Notably, these MAGs displayed an abundance of genes conferring resistance to heavy metals and other abiotic stresses, particularly those affiliated with the keystone taxa. These genes were found to combat excessive accumulation of zinc/manganese, arsenate/arsenite, chromate, nickel/cobalt, copper, and tellurite. Furthermore, the keystone taxa were found to utilize both organic and inorganic energy sources, such as sulfur, arsenic, and carbon dioxide. Additionally, these keystone taxa exhibited the ability to promote vegetation development in re-vegetated mining areas through phosphorus solubilization and metabolite secretion. In summary, our study highlights the metabolic adaptability and ecological significance of microbial keystone taxa in mineral-affected soils. The MAGs associated with keystone taxa exhibited a markedly higher number of genes related to abiotic stress resistance and plant growth promotion compared to non-keystone taxa MAGs.

## 1. Introduction

Nowadays, the security of the soil’s ecosystem and utility has been significantly affected by the accumulation of heavy metals, which has emerged as a major issue in soil contamination. While some metals are essential for plant growth and development, e.g., copper, zinc, nickel, cobalt, but they can be cytotoxic at high concentrations. The toxicity of iron (Fe) and manganese (Mn) is strongly affected by the properties of soils (i.e oxygen content, pH). On the other hand, metals like chromium, mercury, arsenic, and lead are not essential for plants and can be severely toxic even at low concentrations (Tang et al., 2023). The primary cause of toxic metal pollution in the environment is mining operations. Toxic mineral deposits build up around mining and smelting plants may cause significant risks to the ecosystem and public health. An approximately seventy billion tons of wastes are generated by mining each year, of which 14 billion tons are fine particulate tailing matters (Jones and Boger, 2012). Because of the enormous amount of refuse produced, mining wastes are regarded as a significant contributor of environmental pollution through sub-terrestrial leaking, storm-water overflow, and atmospheric deposition (Tordoff et al., 2000). Owing to improper administration, derelict mining deposits have attracted special notice (Dold, 2008). Through stabilizing the mining dumps and lowering subsurface discharge and the aerial dispersion of toxic elements, re-vegetation of mining waste integrated with plant growth-promoting bacteria (PGPB) arbitrarily or naturally—presents a viable option to reduce the ecological effect of mining wastes (Ojuederie and Babalola, 2017; Ashraf et al., 2019).

Microbial community, a significant component of soil, plays a crucial role in the immobility and natural circulation of elements. It is also an important indicator for assessing the ecological effects of toxic metal contamination in the soils (Kumar et al., 2008). Previous studies have shown that the stress of contaminants like heavy metals can decrease the biomass and biodiversity of ecosystems and impact their composition and functionality (Kamal et al., 2010; Zhang et al., 2022). A potential ecologically sound and feasible solution for toxic metal decontamination is bio-remediation. Native microbes are also readily available bio-remediators around polluted locations. These microbes, naturally present in the soil, are capable of removing or transforming heavy metal contaminants into less harmful forms (He et al., 2009; Emenike et al., 2018; Liu et al., 2019; Ma et al., 2019; Li M. et al., 2020). Recent advancements in sequencing technologies have greatly contributed to the study of microbial populations in different habitats. Consequently, there is considerable interest in understanding the changes in microbial community composition and performance caused by heavy metal pollution (Feng et al., 2018; Akash et al., 2022).

Molecular transport mechanisms that facilitate the removal of hazardous metalloids from the cells, metabolic processes that transform metallic ions into less toxicforms, the development of more resilient cell walls and membranes as protection against metalloid toxicity—microbes have employed these intricate strategies to combat the detrimental effects of heavy metals, which can be acquired through the horizontal gene transfer (HGT) process (Li et al., 2019). Microbes are also important for regulating the fates of metalloid, such as speciation (Zhu et al., 2018; Park et al., 2021), solubilizing (Li et al., 2021), and migratory abilities (Lan et al., 2021), in addition to mineral weathering. Studies have determined that the predominant microbial species in areas subjected to long-term thallium pollution are Fe/Mn reducers and Fe/S oxidizers (She et al., 2022). Similarly, in antimony-contaminated streams, *Geobacter* and *Acinetobacter* are the most prevalent microbial species (Sun et al., 2016).

Studies on the spatiotemporal characteristics and successions of native microbial communities in regions with heavy metal contamination have contributed to the selection of potential treatment methods for mining and smelting-intensive areas. One study, for instance, found that *Proteobacteria* was the dominant phylum in all depth layers of heavy metal contaminated soils (Li S. et al., 2020). Understanding the response of microbial communities to metal pressure necessitates a comprehensive description of the ecological processes that regulate the assembly of these communities in environments contaminated with metalloids. Two ecological processes, determinism and stochasticism, are well-known principles that regulate the assembly of microbial community (Shi et al., 2018). Additionally, our previous study also found that the presence of heavy metals significantly impacts the diversity and composition of the microbial communities. Moreover, the community assembly of these communities process becomes more deterministic as the concentration of heavy metals increases. Notably, the proportions of heavy metal-tolerant microbial species, such as *Thiobacillus, Euryarchaeota*, and *Crenarchaeota*, have shown an increase (Kamal et al., 2010; Zhang et al., 2022).

Although the dominant groups and their geographic distributions in circumstances related to metal extraction and smelting have been extensively investigated, there are still gaps in our understanding of the ecological and regulatory mechanisms controlling microbial communities in sites with varying levels or types of metallic ions contamination. The use of genome-resolved metagenomics has increased our understanding of the genetic capabilities of microflora. This study retrieved public sequencing datasets of metagenome soil samples from sites contaminated with heavy metals or rich in metals from the National Center for Biotechnology Information (NCBI) database. Radionuclide (^137^Cs, ^210^Pb, ^226^Ra, ^228^Ra, ^60^Co, ^241^Am, ^238^U, ^228^Th, and ^232^Th) of high levels ranging from 30 to 3,750 Bq/kg and toxic metal (Co, Ni, Cu, Zn, As, Cd, Hg, Pb and Cr) of high levels ranging from 60 to 2,500 mg/kg soil are present in the tested soil samples (Rogiers et al., 2021). To explore microorganisms in metal-contaminated soils and identify potential candidates for bioremediation, the metabolism capacities of the microbial population residing in the metal-rich location were examined using metagenomic profiling. Additionally, metagenomic binning was employed to generate metagenome-assembled genomes (MAGs) and analyze the genetic profiles of metal resistance and transformation in the native microbes, particularly the keystone species. Our findings highlight the important role of the keystone species residing in the re-vegetated deposits, and deliberate managements of such microbiota may contribute to tailing restoration techniques. By examining the metabolic potency of significant microbiota, specifically keystone taxa, in understanding key natural processes involved in mining waste restoration, this study offered a novel perspective. Our research suggests that microbes such as *Burkholderiaceae, Pseudomonadaceae* and *Xanthobacteraceae* can transform different metals by modifying their transportation and cytotoxicity, potentially providing significant ecological benefits such as promote vegetation.

## 2. Results

### 2.1. Metagenome analysis and binning

**Figure 1:**
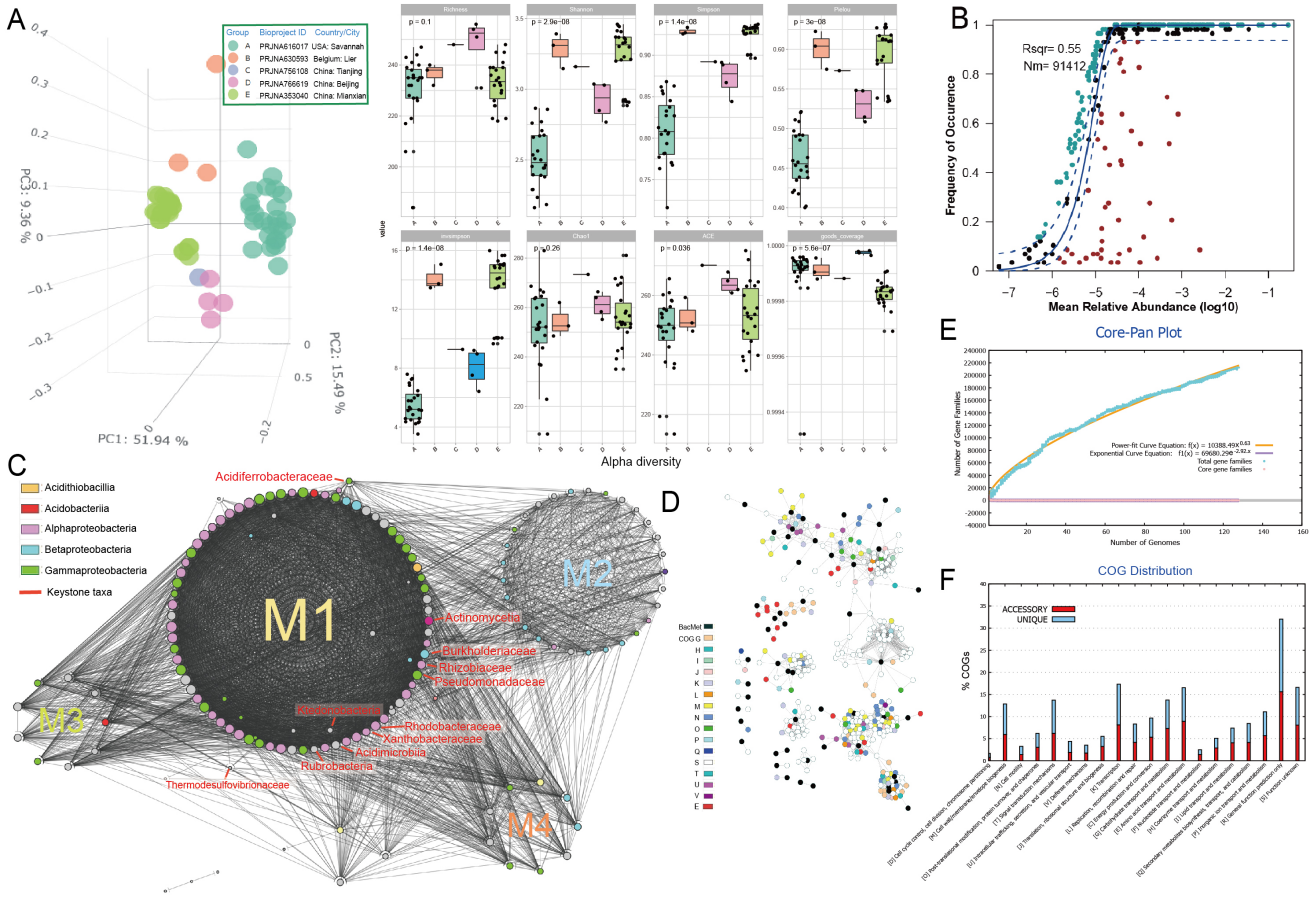
Microbial community analysis based on metagenome data of heavy metal-affected soils. **(A)** Left, Principal component analysis (PCA) of Bray–Curtis dissimilarity matrices showing clustering status of community structure based on the numerical distributions of microbial taxa in tested metagenomes [group **(A–E)**]; right, alpha diversity indexes including Richness, Shannon, Simpson, Pielou, invsimpson, Chao1, ACE, and goods coverage of microbial communities in each group **(A–E)**. **(B)** Fit of the neutral community model (NCM) of community assembly. The OTUs more frequently present than predicted are in cyan, whereas those less frequently are in red. The blue dashed lines represent 95% confidence intervals around the model prediction and the OTUs fallen into the confidence intervals are regarded as neutrally distributed. Nm indicates the meta-community size times immigration, and Rsqr indicates the fit to the neutral model. Neutral processes are the part within 95% confidence interval (red) while non-neutral are the parts including above and below prediction (dark green). **(C)** Molecular ecological networks of microbial community visualized with circular layout in Cytoscape software. Modules (abbreviated as “M”) are separated by different circles. The size of each node is proportional to the degree (number of connections) and the colors of nodes represent different class. The links between the nodes indicate strong (*cor* > 0.8) and significant (*p* < 0.01) correlations. The identified keystone hubs by cytoHubba (Chin et al., 2014) are marked with red-color labels. **(D)** The sub-network containing genes annotated as BacMet (database of antibacterial biocide- and metal-resistance genes) entries and their first neighbors extracted from the gene co-occurrence network constructed across MAGs. The nodes are colored by their annotated function classes. Links between the nodes indicate strong (*cor* > 0.8) and significant (*p* < 0.01) gene co-occurrence in MAGs. **(E)** Pangenome analyzes and mathematical modeling of the size succession of the pangenome. **(F)** Bar chart showing functional proportions (based on COG categories) of different parts of the pangenome (i.e., accessory, unique).

To investigate the metabolisms of key microbial community members that may be involved in the primary succession of the mineral-affected terrestrial areas, metagenome analysis was conducted on soil samples of metal-rich areas (*n* = 58, see Supplementary Table S1 at https://doi.org/10.6084/m9.figshare.22579942.v1). The taxonomic annotation revealed a predominance of phyla *Actinobacteria, Proteobacteria* and *Acidobacteria* in the tested contaminated soil metagenomes (Supplementary Figure S1), which is consistent with previous studies (Lin et al., 2022; Ma et al., 2022). Principal component analysis (PCA) based on the numerical distributions of microbial taxa in tested metagenomes showed that the metagenomes were significantly differentiated among samples. The three main axes explained a total of 76.8% variation (Figure 1A, left, analysis of similarity [ANOSIM] R = 0.854, *p* = 0.001). Metagenomes of bioproject PRJNA616017 (group A) from America have relatively lower alpha diversity indexes (i.e., Shannon, Simpson, Pielou, and invsimpson) compared toother samples (Figure 1A, right). The neutral community model (NCM) predicts approximately 55% of the variability in microbial community taxonomic occurrence frequency can be attributed to relative abundance, indicating a slight dominance of stochastic processes in community assembly (Figure 1B). Different enriched taxa were also identified (Supplementary Figure S2). For instance, plant-associated bacteria such as *Phyllobacteriaceae, Burkholderiales, Pseudomonadaceae, Rhodobacteraceae, Rhizobiaceae, Sphingomonadaceae, Methylobacteriaceae,* and *Actinomycetia* were found to be more abundant in the rhizosphere soil type, specifically in PRJNA766619 samples (Geng et al., 2022). Conversely, soil samples from the river basin, PRJNA630593 (Rogiers et al., 2021) and PRJNA616017 (Thomas et al., 2020), exhibited a higher abundance of nitrogen-transforming microbial taxa, such as *Nitrobacteraceae*, *Nitrosomonadales* and *Thaumarchaeota.* Additionally, redundancy analysis (RDA) indicated that the soil microorganisms displayed different responses to the types of metals. For example, *Nitrobacteraceae* demonstrated a positive correlation with the concentrations of arsenic and lead, but a negative correlation with other metals. Conversely, Burkholderiales exhibited the opposite trend.

**Figure 2:**
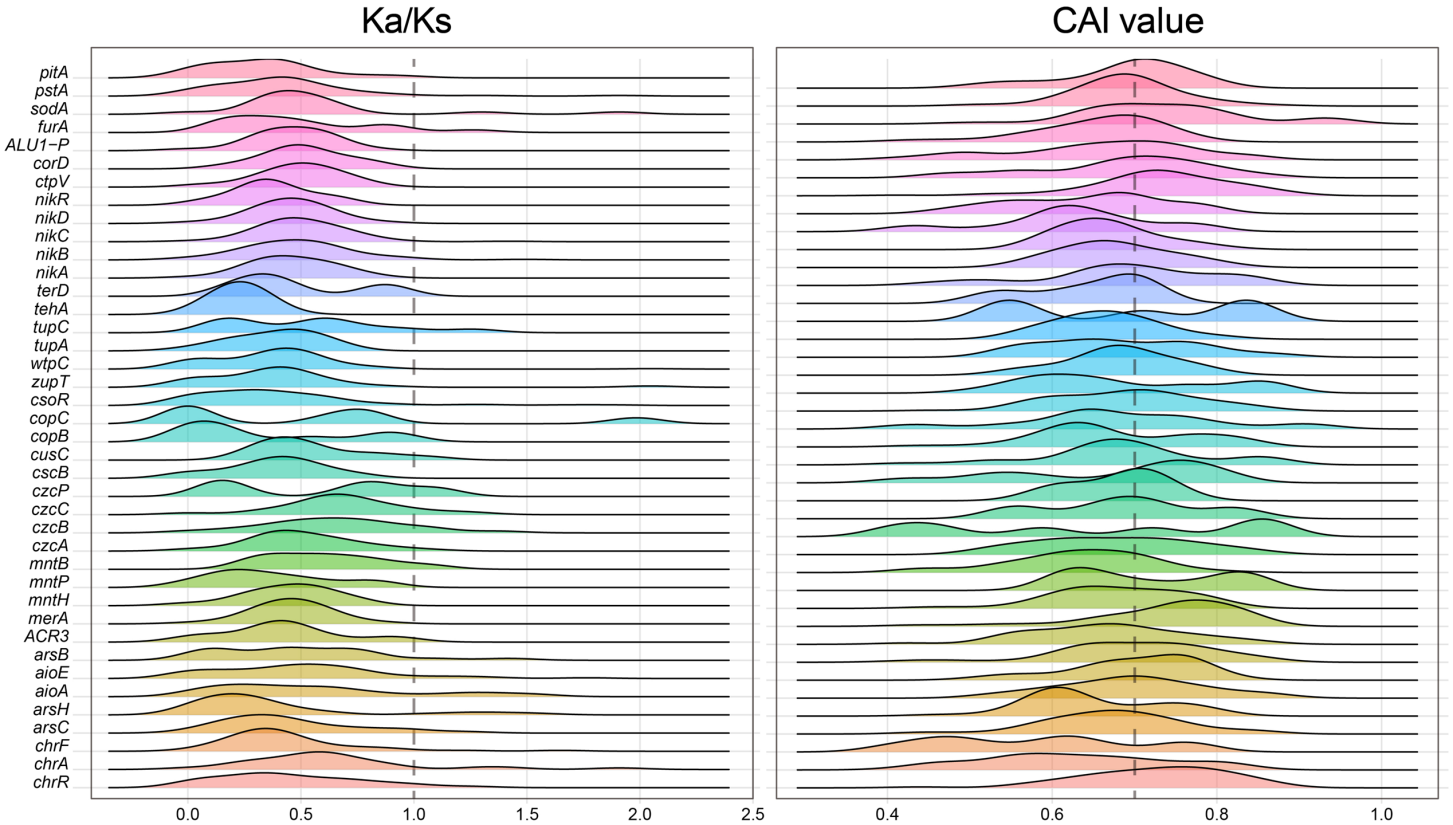
Distribution of Ka/Ks ratio (left) and CAI values (right) measuring the natural selection pressure and synonymous codon usage bias (the higher the CAI value, the higher the expression) for the heavy metal resistance genes classified by BacMet annotation, respectively.

The constructed microbial ecological network revealed four main modules, with the identification of *Burkholderiaceae*, *Rhizobiaceae*, *Xanthobacteraceae*, *Pseudomonadaceae* and other taxa as keystone hubs (Figure 1C, with labels marked with red color) by the software cytoHubba (Chin et al., 2014). After metagenome binning, a total of 175 metagenome-assembled genomes (MAGs) of medium quality or above (completeness >50% and contamination <10%) are recovered, in which 31 MAGs are of relatively high quality (completeness >80% and contamination <10%), and 73 MAGs were assigned to putative novel taxonomic level above genus (see Supplementary Table S2 at https://doi.org/10.6084/m9.figshare.22579942.v1). Pangenome mathematical modeling of the retrieved 175 MAGs revealed an “open” pangenome fitted into a power-law regression function [Ps (x) = 10388.49x^0.63^], while the core genome was fitted into an exponential regression [Fc (x) = 69680.29 e^−2.92x^] (Figure 1E). These results indicate vast genetic diversity within the microbiota from heavy metal contaminated soils and that the currently characterized features of the soil-borne microbial MAGs are still far from saturation. Functional annotation based on clusters of orthologous group (COG) revealed that the accessory genome of tested MAGs had a higher proportion of genes classified in COG categories such as K (Transcription), M (Cell wall/membrane/envelope biogenesis) and L (replication, recombination, and repair) (Figure 1F), which were probably related to environmental adaptions. Within the retrieved MAGs, 50 MAGs are related to the keystone taxa, such as *Burkholderiaceae* (*n* = 9), *Rhizobiaceae* (*n* = 8), *Xanthobacteraceae* (*n* = 4) and *Pseudomonadaceae* (*n* = 1). Still, we failed to retrieve MAGs of some keystone taxa, such as *Acidiferrobacteraceae*. We further constructed the gene co-occurrence network across the high-quality MAGs (Supplementary Figure S3) and extracted the sub-network containing genes annotated as BacMet entries (database of antibacterial biocide- and metal-resistance genes) (Pal et al., 2014) and their first neighbors (Figure 1D). We observed that toxic metal resistance genes (annotated as BacMet entries) co-occurred with the genes of COG classes E (Amino acid transport and metabolism), G (Carbohydrate transport and metabolism), U (Intracellular trafficking, secretion, and vesicular transport), V (Defense mechanisms), M (Cell wall/membrane/envelope biogenesis) and others. This finding is consistent with the consensus that microbial heavy metal stress response involves not only specialized metal transporters but also various cellular processes associated with systems-level maintenance (Pal et al., 2022). Accordingly, multiple gene families associated with heavy metal resilience, environmental adaption and plant growth promotion were identified from the MAGs (see Supplementary Table S3 at https://doi.org/10.6084/m9.figshare.22579942.v1), which will be discussed in the following sessions. Additionally, we performed selection pressure and codon adaption index (CAI) analyzes, indicating that nearly all metal resistance related genes that MAGs encoded exhibited a Ka/Ks ratio below the cutoff value 1 and relatively high CAI values (with average 0.70) (Figure 2).

### 2.2. Metal resistance and defense system

**Figure 3:**
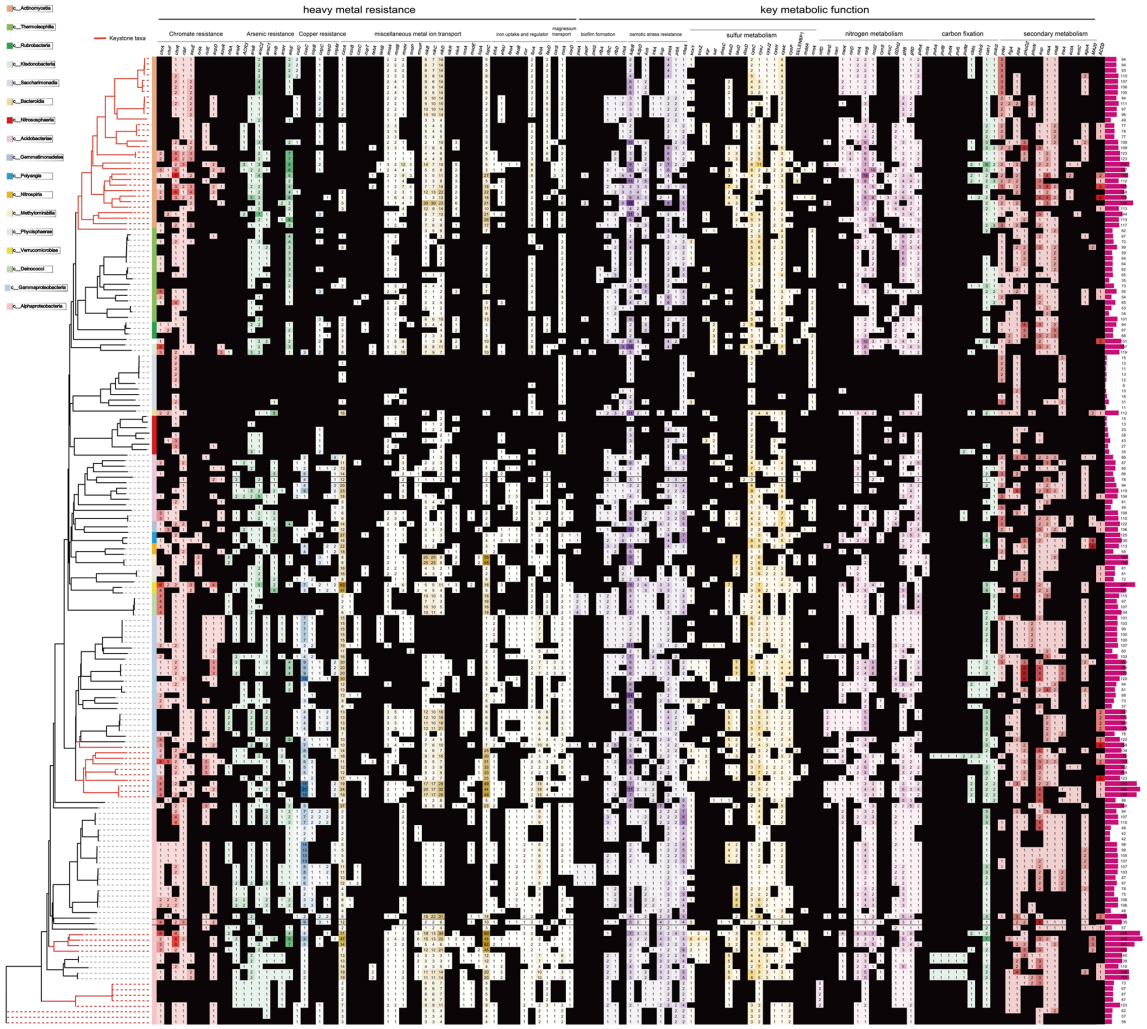
Heatmap showing distribution of gene families related with metal resistance, environmental adaption and metabolic function among tested MAGs. The taxonomic details are shown in the label. Branches leading to keystone taxa MAGs are marked in red color. The detailed description for gene abbreviations is provided in Supplementary Table S3 at https://doi.org/10.6084/m9.figshare.22579942.v1.

The majority of the *Proteobacteria* and several *Acidobacteriota*-associated MAGs (*n* = 93, 56%) contained the Cr(VI) transporter-related genes (*chrAH*), and the best-studied Cr(VI) reductase encoding gene, *chrR*, was observed in almost all reconstructed MAGs (*n* = 126, 73.0%). This includes the MAGs classified into archaea *Nitrososphaeraceae*. Other genes that may be involved in Cr(VI) reduction, including the FMN reductase *ssuE* (*n* = 49, 31% MAGs), FMN-dependent NADH-azoreductase *acpD/azoR* (*n* = 64, 39% MAGs), 3-hydroxypropanoate dehydrogenase *rutE* (*n* = 64, 39% MAGs), nitroreductase *nfsA* (*n* = 64, 39% MAGs), riboflavin kinase/FMN adenylyltransferase *ribF,* and FMN reductase *nrfA* (Kwak et al., 2003; Mugerfeld et al., 2009; He et al., 2010; Zheng et al., 2015; Pradhan et al., 2016), were also annotated (Figure 3).

**Figure 4:**
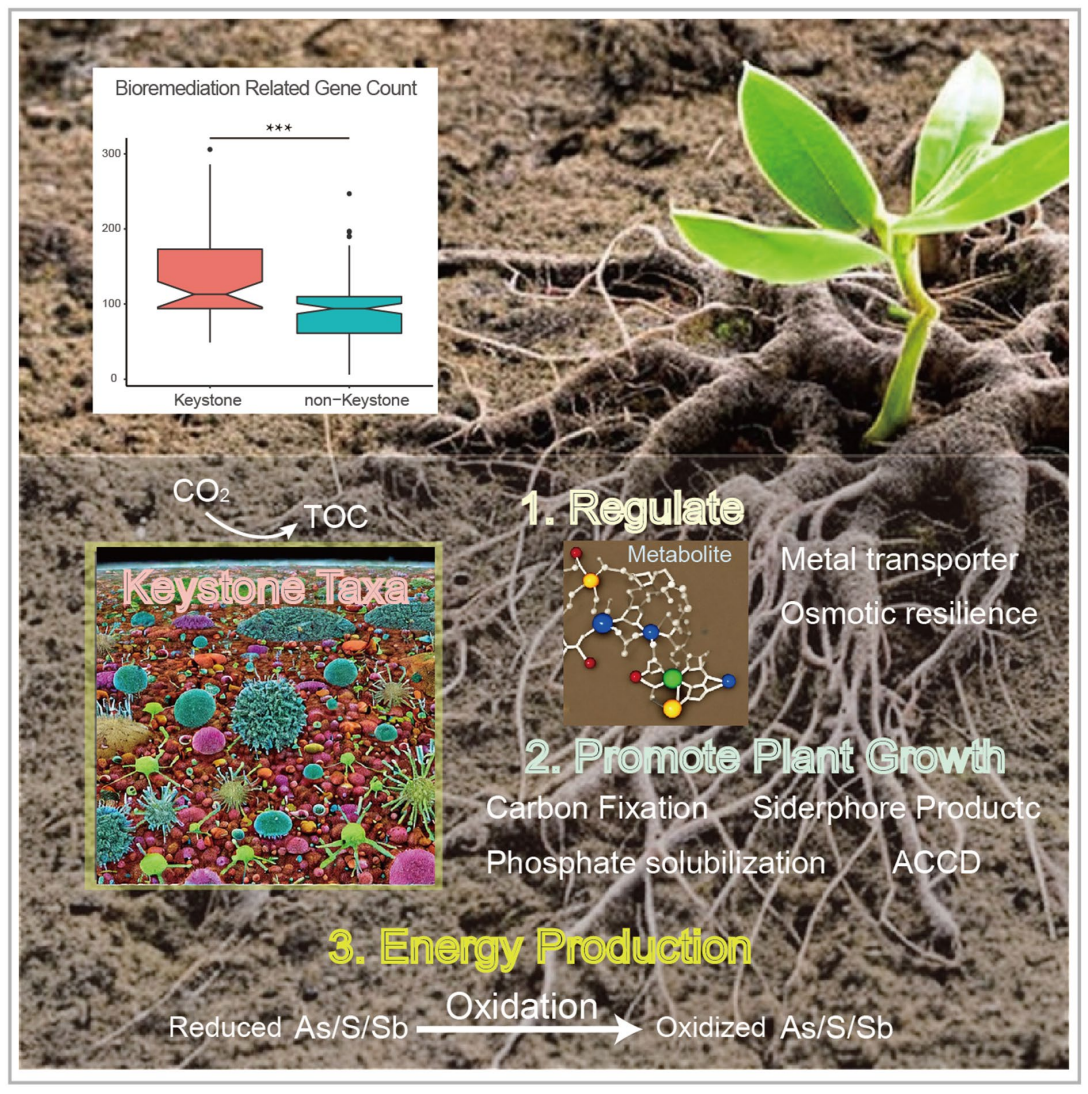
Comparison of the bioremediation-related gene amount between the keystone taxa MAGs and non-keystone MAGs (top, with the asterisks indicate significance; ***, *p* < 0.001), and the conceptual model of the ecological roles conducted by the keystone taxa within heavy metal contaminated soils (bottom).

The majority of the MAGs contained two putative genes *arsC*, which encodes the enzyme arsenate reductase, and *arsR,* which encodes the - transcriptional repressor responsive to arsenate, arsenite and antimonite. However, these genes were not found in MAGs belonging to *Patescibacteria*. The ACR3 gene, responsible for transporting As(III), was found in approximately 23.8% of the MAGs (Figure 4). The *arsB* gene, encoding the arsenite transporter, was also prevalent, present in 58% of the recovered MAGs. The *arrB* gene, which encodes a putative respiratory As(V) reductase involved in As detoxification, was detected in 29 MAGs (21%), mainly found in *Acidobacteriota*. In contrast, the *aioE* gene encoding putative As(III) oxidases (Wang Q. et al., 2017), was only found in one MAGs. Another arsenic resistance protein, ArsH, which acts as a methylarsenite oxidase, was also found encoded by 32 MAGs (17%), predominantly in *Gammaproteobacteria* and *Alphaproteobacteria*. This protein is an organoarsenical oxidase enzyme responsible for conferring resistance to methyl As(III) derivatives in both *P. putida* and *S. meliloti* (Páez-Espino et al., 2015).

A considerable number of putative genes encoding proteins implicated in diverse toxic ion transport and detoxification were found. These included the copper resistance system encoded by *copABCDM/cutC/cusC*, which was present in approximately 43% of the MAGs. Additionally, the cobalt-zinc-cadmium efflux system encoded by *czcABCP* was found in around 16% of the MAGs, and the zinc/manganese transport system encoded by *mntABHP/znuB* was identified in approximately 67% of the MAGs. The tellurite resistance system encoded by *tehAB/terADZ* was detected in about 10% of the MAGs. The fluoride exporter crcB, which was present in 68% of the MAGs, the ALU1-P gene for aluminum tolerance, found in 53% of the MAGs, and the peptide/nickel transport system encoded by *nikBCDR*, detected in around 10% of the MAGs, were also observed. Moreover, the nickel/cobalt transporter *nixA*/*rcnA*, the molybdate transport system *modE*, the ferrous iron transport system *feoAB*, and the storage bacterioferritin bfr were present in 29% of the MAGs. The tungstate transport system proteins *tupAC* were found in 6.9% of the MAGs, while the magnesium transporter *corSAD* was present in approximately 84% of the MAGs. Four MAGs contained the mercuric ion transport gene *merT*. Additionally, genes involved in biofilm formation, including *rfbACD*, *pelAFG*, and chitinase, were identified in 60.7, 13, and 13% of the MAGs, respectively (see Supplementary Table S3 at https://doi.org/10.6084/m9.figshare.22579942.v1).

Besides, components of the microbial adaptive immunity systems against exogenous DNA insertion, the CRISPR (Clustered Regularly Interspaced Short Palindromic Repeats)-Cas (CRISPR-associated proteins) systems, including genes encoding CRISPR-associated exonuclease Cas2, Cas3, Cas4 and Cas5d, together with accessory proteins, such as Cmr6, Csd1, Csd2, Csm1, Csm3, Csm5 and Cst2 were detected in several MAGs belonging to *Acidobacteriota* (see Supplementary Table S3 at https://doi.org/10.6084/m9.figshare.22579942.v1), which might be further employed for gene editing in microbes or plants (Fasani et al., 2018).

### 2.3. Resistance to other environmental stresses

A number of genes responsible to resist other abiotic environmental stresses (e.g., osmotic, acidic) were also observed in most MAGs (Figure 3, see Supplementary Table S3 at https://doi.org/10.6084/m9.figshare.22579942.v1). These genes encoded for the potassium transporting system (i.e., *kdpBD*, 96% MAGs; *kup*, 55% MAGs; *kch*, 37% MAGs; *pch*, 36% MAGs; *trkA*, 29% MAGs), phosphate transport system *pst/pit* (83% MAGs). Besides, genes encoded for organic acids degradation (e.g., acetyl-CoA synthetase, 87% MAGs), and Na^+^:H^+^ antiporter *nhaA* (82% MAGs) and pathways for arginine synthesis (e.g., arginine deiminase *arcA*, 39% MAGs, argininosuccinate lyase *argH* and argininosuccinate synthase *argG*, 82% MAGs) which produce ammonia as a by-product for acidic pressure resistance (Shek et al., 2017; Suryaletha et al., 2019) were also found.

### 2.4. Metabolism potentials

In addition, a list of genes involved in the energy metabolism, biosynthesis of diverse secondary metabolites and xenobiotics biodegradation and metabolism was identified in the MAGs (Figure 3, see Supplementary Table S3 at https://doi.org/10.6084/m9.figshare.22579942.v1). Sulfur metabolism genes were widely identified, including those encoding sulfur-oxidizing protein (*soxYZ,* 14% MAGs), sulfide:quinone oxidoreductasewere (*sqr,* 24% MAGs), sulfate adenylyltransferase (*sat*, 19% MAGs), DMSO reductase (*dmsC*, 9% MAGs), alkanesulfonate monooxygenase (*ssuD*, 39% MAGs), thiosulfate dehydrogenase (*doxD*, 39% MAGs), taurine dioxygenase (*tauD,* 38% MAGs) and finally, sulfate transporter and reductase (*cysCHJKP,* 73% MAGs). Likewise, nitrogen fixation (i.e., *nifD*, 7% MAGs, mostly in the genus *Pararhizobium*) and nitrogen metabolism pathways (e.g., *nirABD, narGIK, ncd2, cynS, arc*) were also annotated. Ribulose-bisphosphate carboxylase (*cbbLS*), carbonic anhydrase (*cah*) and phosphoenolpyruvate carboxykinase (*pckA*) for carbon fixation were encoded by ~40% MAGs (mostly *Actinomycetia, Gammaproteobacteria* and *Alphaproteobacteria*). Also, five *Beijerinckiaceae* MAGs (5%) harbor genes encoding photosynthetic reaction center and light-harvesting complex components, including *puhA, pufA, pufB, pufL* and *pufM.* Xenobiotics biodegradation and metabolism genes, including those involved in the degradation of benzoate (i.e., *pcaBCDHIJ*, *bcrBCD, boxC, ligABJ, catCE, galB, badAFH, xylEH*), fluorobenzoate (i.e., dienelactone hydrolase family), chloroalkane and chloroalkene (i.e., haloacid/ haloalkane dehalogenase), steroid (i.e., *hsaAC, choD, cyp125*), nitrotoluene (i.e., *hyaAB, nfsA*), and aminobenzoate (i.e., *nagH, mdlC, desB*). Other genes related to secondary metabolism were also identified. For example, bacteriocin-related genes (e.g., *ydeI*) are detected in 74% MAGs; metabolism genes for compatible solutes such as trehalose (e.g., *ostAB, treA*) and ectoine (e.g., *ectAC*) are detected in 71 and 5% MAGs, respectively; genes for phosphate solubilization (e.g., *phoND, ppa*) are detected in 66% MAGs; genes for siderphore biosynthesis (e.g., *frgA*) are detected in 25% MAGs; and the gene encoding 1-amino cyclopropane-1-carboxylic acid (ACC) deaminase was detected in 20% MAGs. Detailed information of these metabolism genes could be found in the Supplementary Table S3 at https://doi.org/10.6084/m9.figshare.22579942.v1.

## 3. Discussion

In this study, we analyzed metagenome datasets from soils contaminated with heavy metals that were sampled from plant rhizosphere or naturally mineral areas. The main objectives were to investigate the microbial diversity, metabolic potential,and distribution. Additionally, the microbial correlation network assisted in identifying and gaining a deeper understanding of the diverse key microbes that likely participate in mining waste restoration. Comparing the metagenome-assembled genomes (MAGs) of the keystone taxa to the non-keystone taxa, we observed that the former had more genes related to abiotic stress resistance and promotion of plant growth, as confirmed by an unpaired t-test (*p* < 0.05). Based on these findings, we propose a conceptual model to illustrate the ecological role of the keystone taxa in mineral-affected soils (Figure 4).

Restoration and re-vegetation of heavy-metal polluted deposits are essential procedures for mitigating ecological risks (Bai et al., 2023). Significant efforts have been made to remediate and re-vegetate mineral areas such as tailing waste through microbe inoculation, which has shown certain effectiveness. Soil microorganisms play a crucial role in the initial accumulation of necessary ingredients, including hydrocarbon and accessible nitrogen, for other species (Sun et al., 2018). However, the harsh conditions in mineral-affected areas often limit bacterial growth, which negatively impacts remediation and re-vegetation efforts (Bai et al., 2023). Therefore, understanding the capabilities of soil microbes in terms of environmental adaptation and vegetation development is necessary for effective remediation strategies. Native microflora residing in heavy metal-rich soils or tailings is of considerable interest due to their potential ecological significance. The density of keystone species within the ecosystem may influence the composition of the microbial population (Agler et al., 2016). Their absence from the community can lead to significant disruptions in the ecological functions of the environment (van der Heijden and Hartmann, 2016). Given their importance in microbial populations and the natural “small world” phenomenon, the identification of keystone taxa is crucial (Dunne et al., 2002). Within the microbial ecological network, certain members such as *Actinomycetia, Rhizobiaceae, Pseudonocardiaceae, Acidobacteria* (Ventura et al., 2007) and *Burkholderiaceae* play a vital role and are considered keystone species in the community (Figure 1C). The keystone taxa in this study were chosen from the microbial ecological network according to the recently proposed standards of strong connectivity (closeness centrality >0.475) and small betweeness (betweenness centrality <0.025) (Berry and Widder, 2014; Banerjee et al., 2018a,b). These principles have commonly been employed in the identification of the keystone taxa in terrestrial ecosystems (Liang et al., 2016; Li et al., 2017; Banerjee et al., 2018a,b). The genetic composition of these species was analyzed using metagenomic binning analysis to identify species that are important to the remediation and vegetation. Due to their ability to perform crucial ecological functions, keystone taxa could contribute to re-vegetation efforts in areas affected by toxic waste. *Actinobacteria*, a phylum that encompasses members regarded as an ancient bacterial lineage with diverse environmental habitats, were specifically recognized as keystone taxa in extremely oligotrophic conditions such as Antarctic rock and soils (Le Roes-Hill et al., 2009; Hill et al., 2016), so do *Acidobacteria* (Xu et al., 2022). Members of the *Rhizobiaceae, Burkholderiaceae* and *Pseudomonadaceae* families are frequently found as keystone taxa in plant-associated environments (Chiarini et al., 1998). This finding aligns with the discovery of these microbes as keystone species in metal-rich deposits in our study. These taxa possess versatile and flexible metabolic pathways that can aid in plant growth promotion and resistance against pathogens (Jiang et al., 2017; Wang H. et al., 2017; Yan et al., 2017). Further investigation into the metabolic capabilities of such microbes will reveal the ecological significance of these keystone species and their potential in restoring harsh environments, such as heavy metal-contaminated soils or tailings. Our research also showed that MAGs associated with the keystone taxa have the ability to remediate heavy metals and improve nutrient availability for plants, highlighting their importance in ecological restoration. However, more research is required to fully comprehend the underlying mechanisms and develop effective strategies for utilizing these capabilities in environmental remediation.

### 3.1. Heavy metal resistance genes

Various metals can be transformed by microbes to modify their mobility and toxicity, potentially yielding ecological benefits (Giller et al., 2009). Multiple heavy metal resistance genes have been identified in the MAGs. Their functionality were highlighted by high CAI values, indicating ahigh predicted expression level, and low Ka/Ks ratio, indicating strong negative selection (Figure 2). Arsenic (As) is toxic to microbes and exerts significant selection pressure on the soil microbiota (Chen et al., 2020). Potential arsenic-resistance genes were found in recovered MAGs, particularly in the keystone taxa. Notably, genes involved in As detoxification (e.g., *arsR*) and those encoding the As(III) efflux pump (e.g., ACR3) were frequently detected, especially in keystone taxa like *Rhizobiaceae* and *Burkholderiaceae* (Figure 3). This suggests that soil-borne microbes have played a role in resisting the harmful effects of arsenic. Furthermore, the As(V) respiratory reductase encoded by *arrB* gene and arsenate reductase encoded by gene *arsC* are responsible for As(V) reduction. Microbes can generate energy during the redox processes of arsenic. As(V) reduction is likely common among the microbiome in metal-rich soil, as microbe-mediated As recycling could alter the transportation and cytotoxicity of As.

Furthermore, several genes associated with the reduction of Cr(VI) have been identified. The chromate ion [Cr(VI)] is highly toxic to microbes. Microbes have evolved two primary mechanisms to counteract Cr(VI): ion transport (efflux) and Cr(VI) reduction. The protein ChrA, which acts as a chromate transporter, has been discovered in various microbes, and it plays a role in moderating the extravasation of Cr(VI) (Ouertani et al., 2020; Kusumawardhani et al., 2022). Potential *chrA* genes were extensively discovered in the MAGs of *Deinococcus, Ktedonobacteraceae* and keystone taxa *Burkholderiaceae, Pseudomonadaceae*, and *Xanthobacteraceae* (Figure 3, see Supplementary Table S3 at https://doi.org/10.6084/m9.figshare.22579942.v1). This finding suggests that these microorganisms may be involved in the transport of Cr(VI) ions across cell membranes. *Deinococcus*, for instance, is a kind of radio-resistant bacterium that can detoxify Cr(VI), U(VI) and Tc(VII) from soil (Fredrickson et al., 2000). However, the chromate resistance of *Ktedonobacteraceae* has been rarely reported. The reduction of toxic Cr(VI) to Cr(III) by microorganisms has significant microbiological implications since Cr(III) is less bioactive and hazardous as compared to Cr(VI), although it is a thermodynamically stable compound. Several microorganisms, including *Pseudomonas* (Rajkumar et al., 2005), *Bacillus* (Das et al., 2014) and *Arthrobacter* (Mauricio et al., 2010), have been found to reduce Cr(VI). The best-characterized Cr(VI) reducing gene *chrR*, which encodes the hydrophilic, homodimer, NADH-dependent flavoprotein chromate reductase (Ackerley et al., 2004), was also widely found in MAGs. Additionally, diverse metal-resistant genes, such as cobalt-zinc-cadmium efflux systems *czc, cus*, and *cop*, have been detected in the MAGs (Figure 3, see Supplementary Table S3 at https://doi.org/10.6084/m9.figshare.22579942.v1). This suggests that the efflux of metal ions may serve as an important strategy for resisting metal stress. Therefore, metal content may be an important factor influencing the abundances of microbes, in addition to pH. Indeed, many keystone taxa were rich with various metal resistance genes against As, Cr, and Cu in this study. Numerous proteins implicated in diverse toxic ion tolerance and metal transformations were also present in the MAGs. For instance, gene *crcB* that encodes fluoride exporter was detected in 68% of MAGs (Figure 3, see Supplementary Table S3 at https://doi.org/10.6084/m9.figshare.22579942.v1), which may be utilized to counteract the presence of toxic fluoride compounds ubiquitous in the environment (Sonne et al., 2023). Collectively, the detection of genes associated with metal resistance and transformation in these MAGs supports the hypothesis that that the native microbes may alleviate the metal stress in the metal-rich soils.

**Figure 5:**
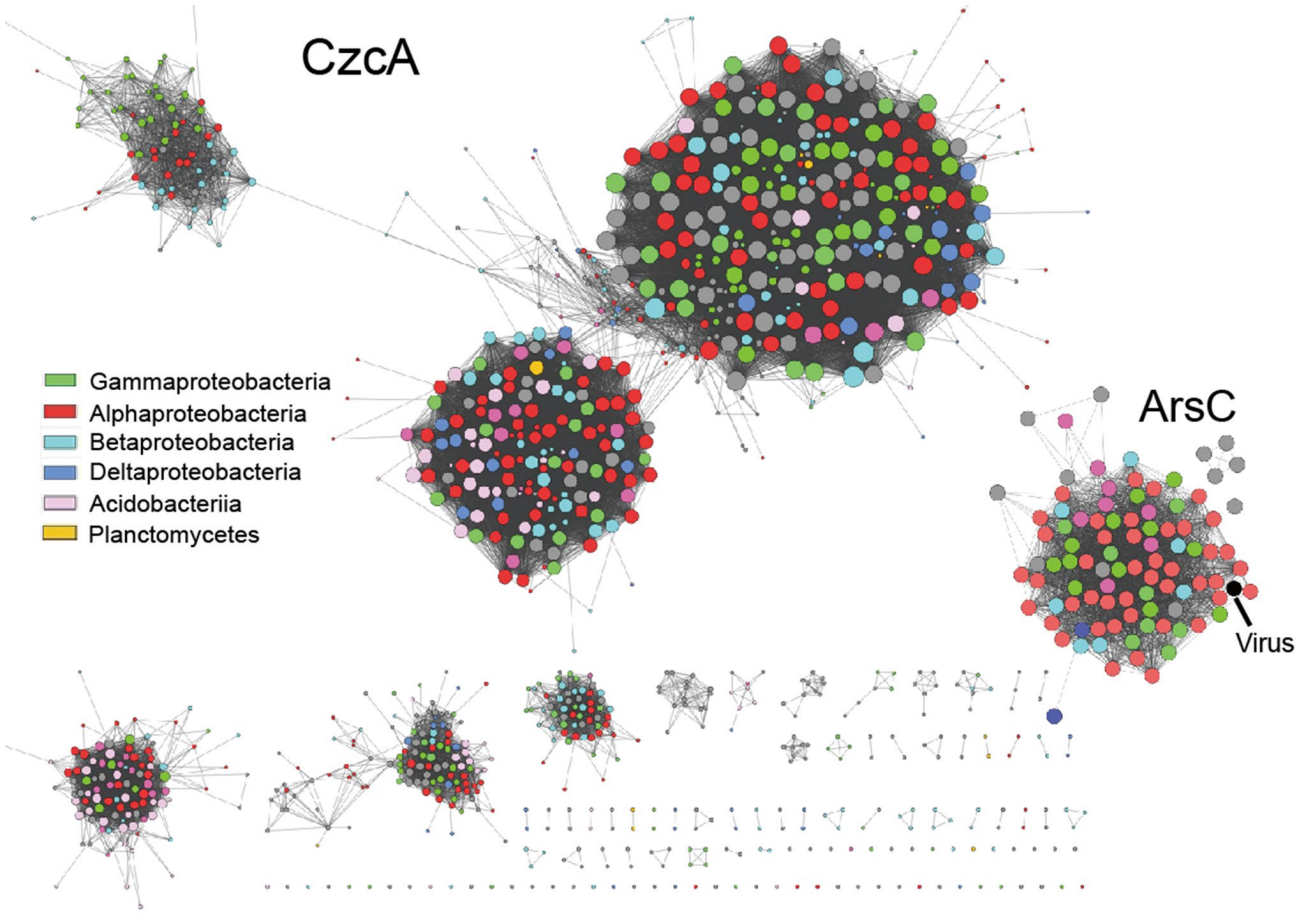
The representative sequence similarity network (SSN) displays heavy metal resistance proteins obtained from metagenome bins that have been tested.

Moreover, we observed a significant cross-linking of phylogenetically distant taxa in the sequence similarity network (SSN) of heavy metal resistance proteins retrieved from tested metagenome bins (Figure 5). This finding indicates that adaptive gene-sharing *via* horizontal gene transfer (HGT), as previously observed in mine drainage environments (Li et al., 2019), is also taking place in our study. Additionally, we have identified heavy metal resistance genes (e.g., *arsC, terCD, copC*) encoded by viral scaffolds recovered from the tested contaminated soil metagenomes (Figure 5, marked with black arrow, see Supplementary data at https://doi.org/10.6084/m9.figshare.22226998.v1). This suggests that the temperate viruses, such as lysogenic bacteriophage, may serve as the vectors for HGT, leading to the accelerated spread of adaptive genetic materials and the increased stress resilience within the microbial community as a whole. Consistent with our previous research, viruses appear to play a crucial role in regulating microbial community assembly (Liu et al., 2023).

### 3.2. Response to other environmental stresses

The survival of microbiota in polluted soils requires resistance to a variety of adverse geochemical conditions, in addition to heavy metals. To maintain a balanced and near-neutral cytoplasm under osmotic pressure, proton buffer mechanisms, such as phosphate-specific transporter (Pst), potassium transporting ATPase system (Kdp), and arginine deiminase (ArcA), Na^+^:H^+^ antiporter (NhaA) are responsible to maintain an equilibrated and near-neutral cytoplasm (Tan et al., 2019). The identification of these associated genes in most of the keystone MAGs (especially *Xanthobacteraceae, Burkholderiaceae* and *Pseudomonadaceae,* as shown in Figure 3) suggests that they facilitate cytoplasmic homeostasis through buffering mechanisms. Specifically, 96% of the microbial genomes were found to possess *kdpBD* genes, which encode the K^+^ transporting ATPase system, indicating the capacity of soil-borne microbiota to relieve osmotic pressure by stimulating K^+^ transporter (Hua et al., 2015). Moreover, the extensive identification of genes encoding acetyl-CoA synthetase (*ACSS*), argininosuccinate lyase (*argH*) and argininosuccinate synthase (*argG*), dTDP 4-dehydrorhamnose reductase (*rfbD*) and others (Figure 3, see Supplementary Table S3 at https://doi.org/10.6084/m9.figshare.22579942.v1) suggests that organic degradation, polyamine production, formation of biofilm and impermeable cell membrane against harmful ions are additional survival strategies for the resistance to osmotic and pH perturbation (Baker-Austin and Dopson, 2007; Suryaletha et al., 2019). These genes may also contribute to the microbial community’s response to heavy metal stress, in line with the consensus that microbial heavy metal stress response involves specialized metal transporters and various cellular processes associated with systems-level maintenance (Pal et al., 2022).

### 3.3. Autotrophic and heterotrophic metabolism of keystone taxa

The verdant site is hypothesized to be aided in tailing replanting by the microflora. Therefore, their metabolic activities are of great interest. Autotrophic organisms play a vital role in environmental restoration as they are the primary producers of organic matter in the environment. They are often present in high numbers during the early stages of restoration. It has been demonstrated that organic matter promotes vegetation development during soil remediation procedures in deposits. In addition to biological carbon, the oxidation of inorganic elements can also impact other geological conditions. For example, oxidation can significantly reduce the movement and cytotoxicity of As. Therefore, the presence of chemolitho-autotrophs in the deposits is essential for environmental recovery. In our study, many keystone MAGs showed the ability to generate energy from inorganic electron donors. For instance, almost all MAGs of keystone *Burkholderiaceae* and *Xanthobacteraceae* contained the sulfur-oxidizing *sox* genes (Figure 3). This suggests that sulfur oxidation may be important for keystone species. This study confirmed previous findings that sulfur oxidation genes and microbial species were highly enriched in the mining-impacted region (Sun et al., 2020). Since metal ores are commonly found in the form of sulfide minerals, tailings often contain high amounts of reduced sulfur compounds. Therefore, energetic production through the oxidation of reduced sulfur molecules may fuel the keystone taxa responsible for carbon fixation in the microbial community. On the other hand, the oxidation of large amounts of arsenic (As) and antimony (Sb) pollutants can also contribute to the flourishing of keystone taxa in metal-rich areas like tailings (Zhang et al., 2020), since the keystone taxa *Rhizobiaceae, Xanthobacteraceae, Burkholderiaceae* and *Actinomycetia* harbor the gene *arsH* or *aioE* (Figure 3).

The energy efficiency, imperative for surviving in nutrient-poor tailing environments, may be influenced by the abundance of carbon fixation genes (Ryu et al., 2003). Previous studies have investigated the functional genes involved in carbon fixation within bacterial life in mineral areas (Sun et al., 2018, 2020). These keystone MAGs may play a crucial role in supplying organic carbon in the re-vegetated mining areas (Schmidt et al., 2008). MAGs of keystone taxa such as *Rhizobiaceae, Burkholderiaceae* and *Actinomycetia* harbor the potency of fixing carbon (Figure 3). Moreover, the microbial ability to degrade xenobiotics, complex organic compounds, and synthesize secondary metabolites aids in their ecological roles within the microflora. The metabolism of essential metabolites can significantly alter the variety and interactions of soil microbes (Coyte et al., 2015; Banerjee et al., 2016). Secondary metabolites refer to a wide range of organic chemicals that interact with other species in soil ecosystems or vegetation, even though they are not necessary for the development or propagation of the microbial species (Coyte et al., 2015; Banerjee et al., 2016; Tyc et al., 2017). For instance, the competition and niche development within the soil’s microbiota may depend on the production bacteriocins (Dobson et al., 2012).

Further investigation was conducted on the capacity of keystone taxa to promote plant growth (PGP), which has potential applications in phytoremediation procedures and supporting vegetation development (Grandlic et al., 2008, 2009). Most of the keystone MAGs such as *Burkholderiaceae*, encoded PGP genes related to siderophore production, phosphate solubilization, and 1-amino cyclopropane-1-carboxylic acid (ACC) deaminase pathways (Figure 3, see Supplementary Table S3 at https://doi.org/10.6084/m9.figshare.22579942.v1). The development of siderophore production and strong iron uptake and retention abilities can provide strategic advantages to microbes during colonization of ecological niches, such as the rhizosphere, and promote vegetation development (Compant et al., 2005). Additionally, the enzymatic hydrolysis of phosphorus and other nutritional compounds promotes vegetation development by converting refractory nutrients like polyphosphate into an ionic form (Rodríguez et al., 2006). Plant growth may be supported by ACC deaminase, which reduces ethylene production in plants (Contesto et al., 2008). The identification of PGP genes in the keystone taxa reinforces previous research findings. For instance, it has been demonstrated that the keystone taxa, *Burkholderiaceae* and *Pseudomonadaceae* produce various auxiliary compounds. *Burkholderiaceae* species, resistant to toxic metals, significantly promote vegetation development by enhancing iron uptake, ACC deaminase activity, and polyphosphate solubility (Jiang et al., 2008). Similarly, metal-resistant *Pseudomonas* strains exhibit these PGP traits in growing vegetation (Loaces et al., 2011; Oves et al., 2013; Hsu and Micallef, 2017). The effective competitiveness and niche maintenance of keystone taxa within soil ecosystems may depend on the production of secondary metabolites like bacteriocins. Consequently, in-depth research on the physiological characteristics of secondary metabolites produced by keystone species may facilitate replanting efforts by regulating the rhizosphere microbiota (Vetsigian et al., 2011). The number of shotgun metagenomes related to metal contaminated soils is still limited compared with the amplicon sequencing data. As sequencing technology advances, future studies will include a wider range of soil samples with metal contamination to confirm the ecological roles of the identified keystone taxa in this study.

## 4. Materials and methods

We first queried and retrieved all the items in the National Center for Biotechnology Information (NCBI) database (Benson et al., 2018) with the keywords “soil” and “metal” within the “biosample” regions. Only “shotgun metagenome” with the total size of clean data over 5GB were retained. This process has filtered the amplicon sequence data that do not provide functional information. We then downloaded from database the out-coming 58 soil metagenomes of microbiome as listed in Supplementary Table S1 at https://doi.org/10.6084/m9.figshare.22579942.v1 for downstream analyzes. Metagenome reads were assembled with MEGAHIT version 1.1.1 with default k-mer options (Li et al., 2015), followed by routine analyzes with the pipeline metawrap v.1.0 (Uritskiy et al., 2018). A combination of metabat2 (Kang et al., 2019), maxbin2 (Wu et al., 2016), concoct (Alneberg et al., 2014) were used for metagenome binning. GTDB-TK (Chaumeil et al., 2022) was applied for taxonomic assignment. Metagenome-assembled genomes (MAGs) quality assessment was conducted by CheckM (Parks et al., 2015) and we categorized the bins into high quality (completeness >80% and contamination <10%) and medium quality (completeness >50 to <80% and contamination <10%) according to a previous study (Tandon et al., 2022). This was followed by protein sequence clustering and analysis through software BPGA v.1.0 (Chaudhari et al., 2016) by default procedures, as well as functional annotation against the eggNOG database version 5.0 (Huerta-Cepas et al., 2019) and BacMet (database of antibacterial biocide- and metal-resistance genes) (Pal et al., 2014). The recovered MAGs in “fasta” format are available at https://doi.org/10.6084/m9.figshare.22579957.v1. A combination of VIBRANT v.2.0 (Kieft et al., 2020) and DeepVirFinder v.1.0 (Ren et al., 2020) were applied for viral scaffold recovery and analyzes under default parameters. The retrieved viral sequences in “gbk” format are available at https://doi.org/10.6084/m9.figshare.22226998.v1.

Codon adaption index (CAI) was used as a numerical estimator of gene expression level (Hiraoka et al., 2009; Zhou et al., 2013), and correspondingly, the webserver CAIcal (Puigbò et al., 2008a,b)^1^ was applied to calculate the CAI values for respective genes. A higher CAI value indicates a higher level of gene expression (Puigbò et al., 2008a,b; Li et al., 2019). We used Ka/Ks Calculation tool^2^ to calculate the ratio of nonsynonymous (Ka) to synonymous (Ks) nucleotide substitution rates is an indicator of selective pressures on metal resistance genes.

To construct the microbe or gene orthologue association network, correlations between pairwise operational taxonomic or gene orthologues that were present in more than half of the samples were calculated using the SparCC (Friedman and Alm, 2012) or CoNet (Faust and Raes, 2016) methods. Only edges with a significant correlation higher than 0.8 (*p* < 0.01) were retained for network construction. Cytoscape v.3.9.1^3^ was used for network visualization. Network topological characteristics were calculated using NetworkAnalyzer tool in Cytoscape. Modular structure of highly interconnected nodes was analyzed using the MCODE application with default parameters. Cytoscape plugin cytoHubba (Chin et al., 2014) with “ClusteringCoefficient” and “BottleNeck” methods was used to predict keystone nodes in the network with the criteria: closeness centrality >0.475 and betweenness centrality <0.025 (Banerjee et al., 2018a,b). To determine the potential importance of stochastic processes on community assembly, the neutral community model (NCM) was used to predict the relationship between microbial taxa detection frequencies and their relative abundance across the wider meta-community, performed using R (version 3.6.3). The NCM is a validated method for deducing stochastic processes related to community assembly, which has been useful in deciphering ecological phenomena (Roguet et al., 2015). This model can quantify the significance of undetectable processes that might have a great impact on microbial communities (i.e., dispersal and ecological drift).

## Data availability statement

The original contributions presented in the study are included in the article/Supplementary material, further inquiries can be directed to the corresponding authors.

## Author contributions

LL, DM, and HY conceived and designed the research. LL, TZ, and YL analyzed the data. LL wrote the manuscript. All authors contributed to the article and approved the submitted version.

## Funding

This research was supported by the Key Projects of Science and Technology of Hunan Branch of China National Tobacco Corporation (grants nos. 2021539200340244, 2020530000242025, xj202106, and 202104) and Fundamental Research Funds for the Central Universities of Central South University (no. 2022ZZTS0420). This study received funding from the Key Research and Development Program of Hunan Province and Natural Science Foundation of Changsha. The funder was not involved in the study design, collection, analysis, interpretation of data, the writing of this article, or the decision to submit it for publication.

## Conflict of interest

TZ was employed by Hunan Urban and Rural Environmental Construction Co., Ltd.

The remaining authors declare that the research was conducted in the absence of any commercial or financial relationships that could be construed as a potential conflict of interest.

## Publisher’s note

All claims expressed in this article are solely those of the authors and do not necessarily represent those of their affiliated organizations, or those of the publisher, the editors and the reviewers. Any product that may be evaluated in this article, or claim that may be made by its manufacturer, is not guaranteed or endorsed by the publisher.

## Footnotes

^1^ http://genomes.urv.es/CAIcal/calc.php

^2^ http://services.cbu.uib.no/tools/kaks

^3^ https://cytoscape.org

## Supplementary material

Supplementary Figure S1 Bar chart showing the taxonomic compositions of tested metagenome of mineral-affected soil samples.

Supplementary Figure S2 **(A)** Kruskal-Wallis analysis was performed to assess the distribution of taxa among the sample groups. **(B)** Redundancy analysis (RDA) was conducted to investigate the interaction between metal type and microorganism abundance.

Supplementary Figure S3 The gene co-occurrence network across MAGs was constructed based on strong Spearman correlation (correlation coefficient > 0.8, *p*-value < 0.05). Nodes in the network were colored according to COG class.
