# Supplementary figures and images for: Genome-resolved metagenomics provides insights into the ecological roles of the keystone taxa in heavy-metal-contaminated soils
Source: Front Microbiol. 2023 Jul 21;14:1203164. doi: 10.3389/fmicb.2023.1203164 (PMC10402746; doi:10.3389/fmicb.2023.1203164)

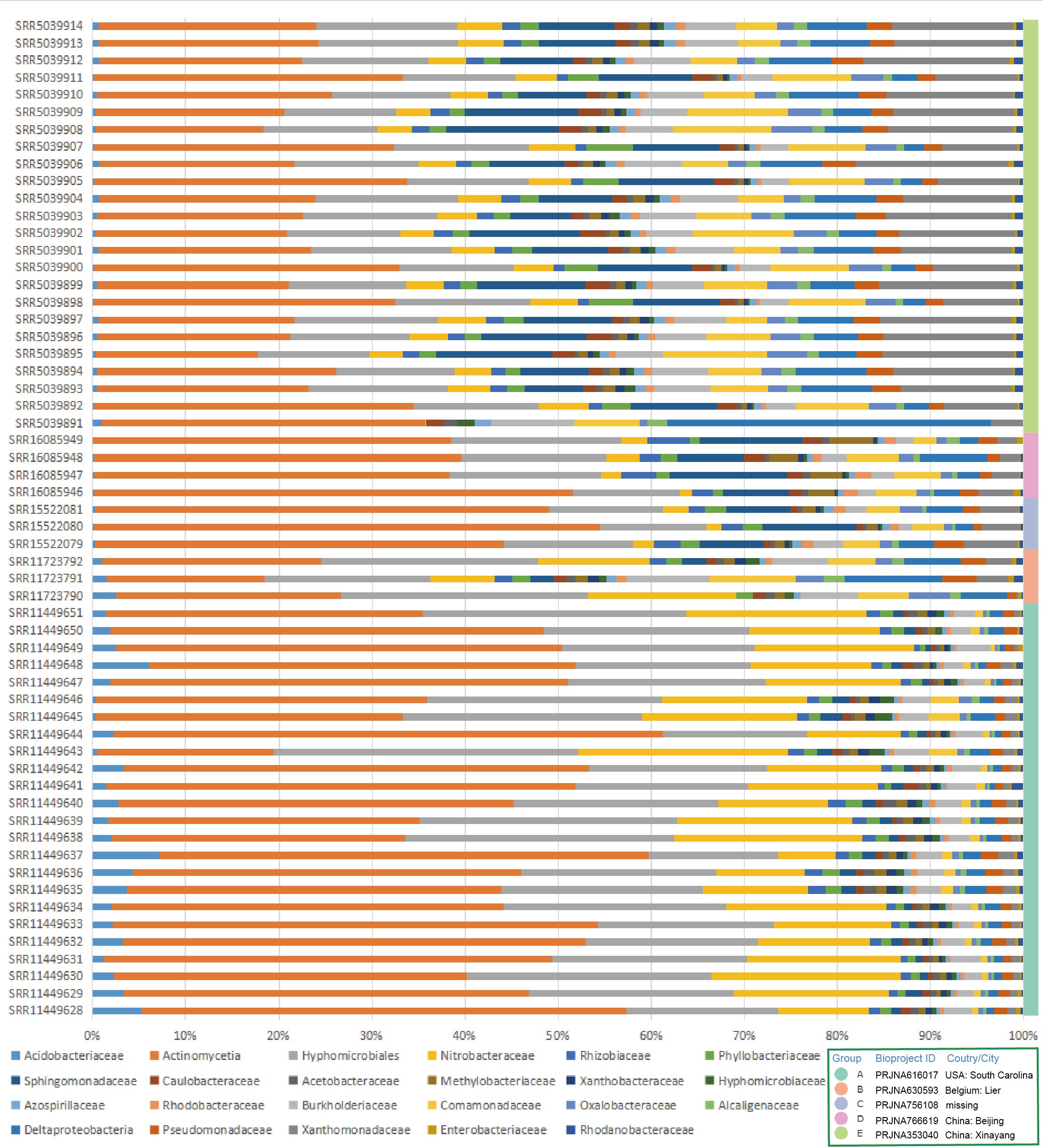

Supplement: Supplementary Figure S1 — Bar chart showing the taxonomic compositions of tested metagenome of mineral-affected soil samples. [file Image_1.JPEG]

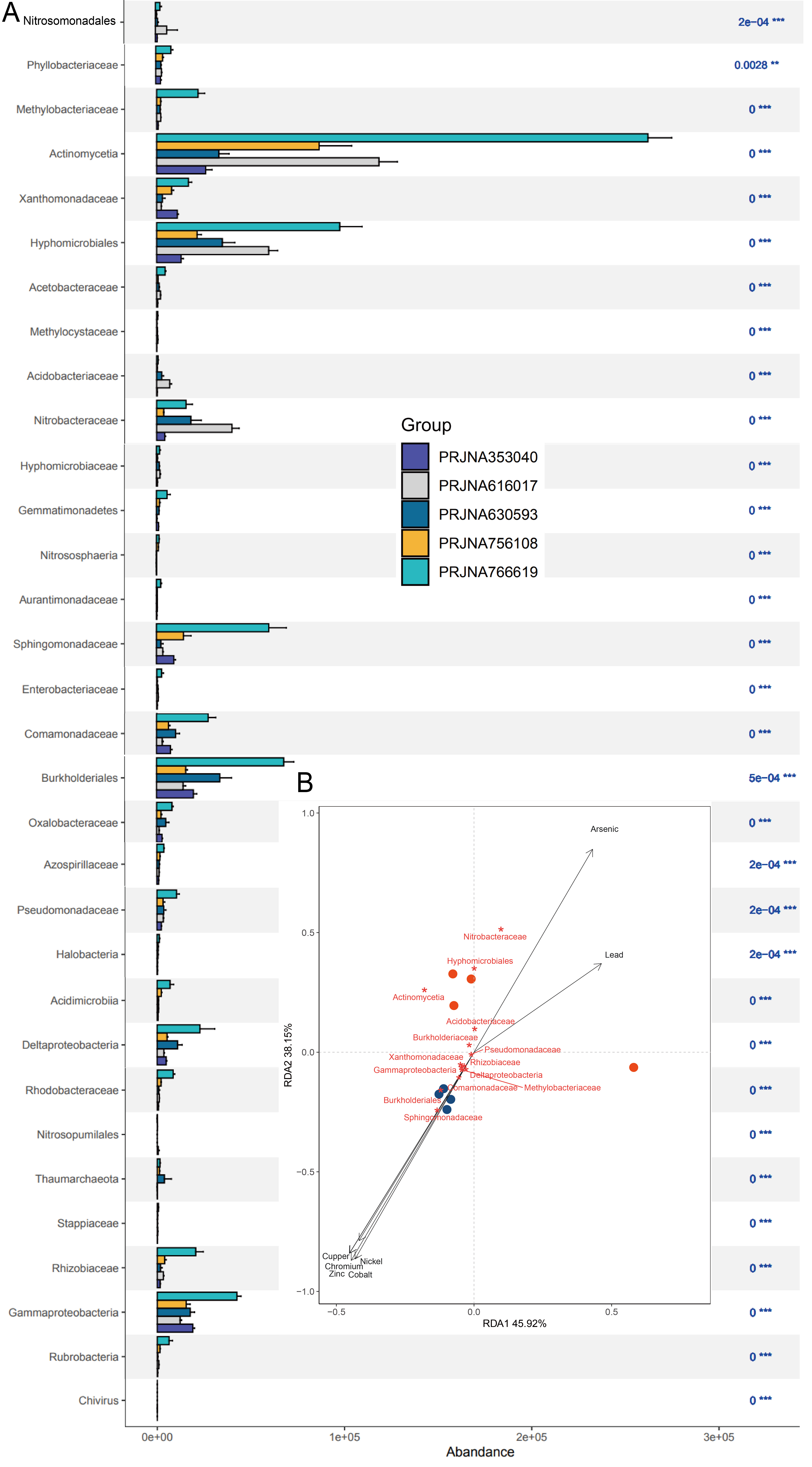

Supplement: Supplementary Figure S2 — (A) Kruskal-Wallis analysis was performed to assess the distribution of taxa among the sample groups. (B) Redundancy analysis (RDA) was conducted to investigate the interaction between metal type and microorganism abundance. [file Image_2.JPEG]

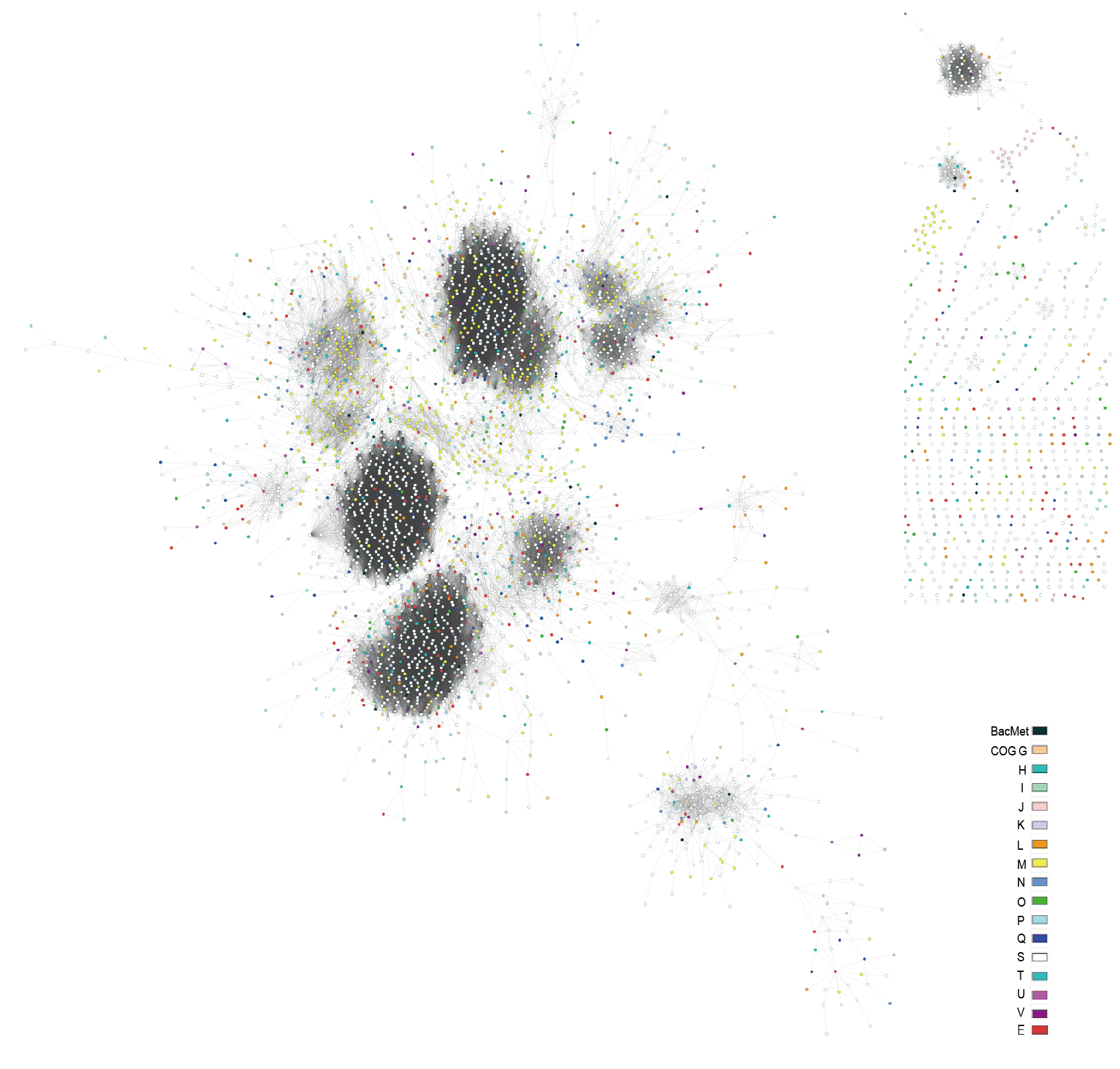

Supplement: Supplementary Figure S3 — The gene co-occurrence network across MAGs was constructed based on strong Spearman correlation (correlation coefficient > 0.8, p-value < 0.05). Nodes in the network were colored according to COG class. [file Image_3.JPEG]
